# Study on suppression of otoacoustic emissions: lateral domain

**DOI:** 10.1590/S1808-86942011000500002

**Published:** 2015-10-22

**Authors:** Jerusa Roberta Massola de Oliveira, Candido Fernandes Fernandes, Orozimbo Alves Costa Filho

**Affiliations:** 1 Master's degree, speech therapist; 2 Full professor, Department of Mechanical Engineering, Paulista State University (Universidade Estadual Paulista). Full professor, Department of Mechanical Engineering, Júlio de Mesquita Filho Paulista State University (Universidade Estadual Paulista Júlio de Mesquita Filho); 3 Full professor, Speech Therapy Department, São Paulo University (Universidade de São Paulo) Bauru campus. Vice-coordinator of the Audiological Research Center (Centro de Pesquisas Audiológicas) São Paulo University (Universidade de São Paulo)

**Keywords:** auditory pathways, otoacoustic emissions, spontaneous, suppression

## Abstract

**Abstract:**

A pon stimulation by contralateral, ipsilateral or bilateral noise, the medial olivocochlear efferent tract changes the amplitude of otoacoustic emissions relative to the tested ear, reducing or removing it; this resulted in a reduction/suppression effect of otoacoustic emissions. Differences in patterns of elimination/reduction of otoacoustic emissions between ears have been documented worldwide; there are, however, no Brazilian studies investigating the effect of lateral dominance.

**Aims:**

To compare the effect of the presence of deletion/reduction of otoacoustic emissions and their amplitude relative to lateral dominance in normal hearing adults.

**Methods:**

A clinical and experimental study. The sample comprised 75 individuals. The methodology was conventional - linear click intensity of 60 dB SPL; white noise was contralateral stimulation at 60 dB SPL.

**Description of results:**

There were no statistically significant differences between right and left ear results, in terms of asymmetry of the degree of otoacoustic emissions and the presence of suppression/reduction.

**Conclusion:**

There is no lateral dominance in the degree of otoacoustic emissions in the presence of suppression/reduction in the study population.

## INTRODUCTION

Suppression of evoked otoacoustic emissions or the efferent olivocochlear reflex is a phenomenon that may be characterized by the suppression of response amplitude or by latency and phase changes of evoked otoacoustic emissions when a contralateral stimulus is presented simultaneously to a recording^1^. Some authors have stated that the suppression effect of evoked otoacoustic emissions may occur when an acoustic stimulus is presented contralaterally, ipsilaterally, or bilaterally to the ear that is being tested at the time evoked otoacoustic emissions are being investigated^2^,^3^. This effect results from activation of the medial efferent olivocochlear tract.

The suppression mechanism of evoked otoacoustic emissions has not been fully clarified; it is thought that the efferent olivocochlear tract in the auditory system operates as a modulator that adjusts active cochlear processes by slow contractions of the outer hair cells, attenuating rapid contractions and specifically generating a protective factor for the inner ear, by means of acoustic stimulation^4^,^5^.

The efferent olivocochlear tract originates around the superior olivary complex. It consists of thick myelinated fibers that project mostly (72% to 74%) to the contralateral cochlea and end directly in the cell bodies of outer hair cells; the remaining fibers (26% to 28%) extend to the ipsilateral cochlea^6^. One of the functions of this tract is to decrease the amplitude of otoacoustic emissions; this is done by a mechanism that modulates slow contractions in outer hair cells based on information transmitted by the afferent auditory system to attenuate rapid contractions. In this manner, the impedance of the system is increased, and these contractions are dampened; this process involves regulation of the length, strain and rigidity of outer hair cells^4^.

The main features of suppression of transient otoacoustic emissions are: it is best studied at a 8 ms to 18 ms amplitude response interval; the amplitude of suppression increases as the intensity of contralateral noise increases; it is stronger for low intensity rather than high intensity stimuli; it is stronger for bilateral rather than ipsilateral or contralateral noise, and for shorter intervals between acoustic stimuli and contralateral noise; it is also stronger for noise lasting more than 400 ms; and, reproducible suppression effects may be seen in a test retest comparison.^1^

Researchers have described differences in suppression/reduction patterns of evoked otoacoustic emissions between the right and left ear. Interaural asymmetry has been found, not only in relation to the amplitude of evoked otoacoustic emissions (it is stronger on the right), but also in relation to the action of the efferent auditory system (also more effective on the right)^7^,^8^.

The activity of the medial efferent olivocochlear tract in both ears of 44 normal hearing subjects aged from 19 to 29 years was studied to compare bilateral inhibition of the tract. Transient evoked otoacoustic emissions (TEOAE) were investigated with clicks at 59 to 71 dB SPL with contralateral white noise stimulation at 30 dB HL. There was more activity of the efferent system on the right; there were no gender differences. This finding could explain the asymmetry of TEOAE - auditory sensitivity is lateralized, and temporary changes in threshold and tinnitus occur. The authors pointed out, however, that further studies are needed to explain such asymmetry^7^.

There are no published studies in the Brazilian literature about lateral dominance of the activity of the medial efferent olivocochlear tract affecting outer hair cells and causing a suppression/reduction effect of otoacoustic emissions, thus, no comparison with international studies is possible. Therefore, the purpose of this study was to compare the suppression/reduction effect of evoked otoacoustic emissions and their amplitude relative to right and left dominance in normal hearing adults.

## METHODS

This clinical experimental trial was undertaken in 2007, and followed the ethical guidelines defined by the institutional review board for research on human beings, which approved the study (number 298/2003-UEP-CEP).

The inclusion criteria were as follows: normal results in audiological testing, and presenting TEOAE bilaterally without contralateral acoustic stimulation. The series consisted of 75 adults of both sexes aged from 20 to 73 years, which were allocated to age groups as shown on Table 1.

**Table 1:** Distribution of the series in this study according to sex and age groups

Group	Total no. of subjects	Sex	
		F	M
1 (20 |- 30)	13	6	7
2 (30 |- 40)	15	11	4
3 (40 |- 50)	15	14	1
4 (50 |- 60)	17	12	5
5 (≥ a 60)	15	12	3

An ILO 292 DP ECHO Research OAE System two-channel device was used to record TEOAE without and with a contralateral acoustic stimulus; two acoustic probes were used. The signal generator issued an acoustic stimulus to evoke otoacoustic emissions (probe A) and issued a contralateral suppressive acoustic stimulus (probe B).

The parameters were: reproducibility (70%), analysis time (2.5 to 20 milliseconds), and frequency range (1 kHz to 5 kHz). The criterion for the occurrence of TEOAE was that a response was present if the amplitude of otoacoustic emissions (in dB) was equal to or higher than 3 dB SPL above noise in at least three consecutive frequencies.

The conventional TEOAE recording mode was used - without and with contralateral acoustic stimulation starting always on the right ear.

The evoking acoustic stimulus in channel A was a linear click at 60 dB pe SPL (± 1) without and with contralateral acoustic stimulation. With contralateral acoustic stimulation, white noise was presented through channel B at 60 dB pe SPL (± 1).

Reduction/suppression may be defined numerically as the amplitude difference (dB NPS) of the otoacoustic emissions response without and with contralateral acoustic stimulation; the value of this difference shows quantitatively the degree of reduction/suppression. Reduction is present when the difference is positive with a decrease in the response amplitude of TEOAE with contralateral acoustic stimulation; suppression is present when TEOAE responses are extinguished. TEOAE reduction/suppression is absent when the difference is zero or negative.

For the purposes of our study, analysis of the presence/absence of the TEOAE reduction/suppression effect was based on the response value, which is the total level of values that correlate specifically with the tracings of memories A and B as analyzed using the fast Fourier transform (FFT); this is considered the true general otoacoustic emissions response after excluding background noise interference. The FFT translates the common response spectrum to memory tracings^9^.

Analysis of variance with repeated measures was applied for the inferential analysis between the response and the factors ear, group, and noise^10^.

## RESULTS

The factor “ear” was checked to see if it was a significant variable; analysis of variance with repeated measures found that this probability was 0.035, therefore statistically significant, as shown on Table 2. Thus, the values for the right and left ears may be compared in the inferential analysis.

**Table 2:** Results of analysis of variance with repeated measures for each factor

Factor	Probability
Group	0,004
Ear	0,035
Noise	<0,0001
Group*ear	0,543
Group*noise	0,036
Ear*noise	0,404
Group*ear*noise	0,128

Key: significance level: < 0.05 %

Annex 1 presents the results of comparing the presence of the suppression/reduction effect of evoked otoacoustic emissions and its amplitude relative to right/left dominance in normal hearing healthy adults. The statistical treatment will be presented below.

**Annex 1:** Results for all subjects

Subject	Number	Group	Gender	Age (years)	Response NN	Response WN	Degree of reduction	Reduction/suppression
RMY (RE)	1				4,1	2,2	1,9	↑
RMY (LE)		1	M	25	5,1	4,3	0,8	↑
TV (RE)	2				11,4	10,2	1,2	↑
TV (LE)		1	F	24	8,0	7,6	0,4	↑
PF (RE)	3				8,3	7,2	1,1	↑
PF (LE)		1	F	29	8,4	8,2	0,2	↑
RS (RE)	4				12,6	12,1	0,5	↑
RS (LE)		1	M	25	4,9	5,0	-0,1	↓
KS (RE)	5				4,1	4,1	0	↓
KS (LE)		2	F	30	3,5	3,9	-0,4	↓
VR (RE)	6				5,6	5,2	0,4	↑
VR (LE)		1	F	24	5,3	4,6	0,7	↑
SCA (RE)	7				9,5	8,0	1,5	↑
SCA (LE)		1	F	26	5,3	4,3	1	↑
CFA (RE	8				3,4	3,4	0	↓
CFA (RE		1	F	26	8,1	9,7	-1,6	↓
KAC (RE)	9				15	13,7	1,3	↑
KAC (RE)		1	F	23	12,6	10,9	1.7	↑
PEC (RE)	10				0,3	0,4	-0,1	↓
PEC (LE)		2	F	30	2,8	0,8	2	↑
MNV (RE)	11				9,5	8,2	1,3	↑
MNV (LE)		1	M	20	8,2	7,4	0,8	↑
RBG (RE)	12				2,8	1,7	1,1	↑
RBG (LE)		1	M	20	4,2	3,8	0,4	↑
JK (RE)	13				9,6	9,2	0,4	↑
JK (LE)		1	F	22	6,7	6,1	0,6	↑
SVM (RE)	14				9,8	9,5	0,3	↑
SVM (LE)		1	M	22	10	10	0	↓
FAO (RE)	15				2,7	2,3	0,4	↑
FAO (LE)		1	M	29	3,9	2,8	1,1	↑
NAN (RE)	16				4,5	2,5	2	↑
NAN (LE)		1	M	38	3,0	0,8	2,2	↑
DDR (RE	17				12,8	11,8	1	↑
DDR (LE)		2	F	33	8,8	8,5	0,3	↑
FZ (RE)	18				7,1	6,0	1,1	↑
FZ (LE)		2	M	35	3,1	2,8	0,3	↑
LCG (RE)	19				5,0	4,3	0,7	↑
LCG (LE)		2	M	37	6,3	4,8	1,5	↑
PAN (RE)	20				1,2	2,3	-1,1	↑
PAN (LE)		2	M	38	0,7	0,4	0,3	↑
MJL (RE)	21				7,5	7,3	0,2	↑
MJL (LE)		2	F	35	8,5	7,3	1,2	↑
ACS (RE)	22				2,7	2,1	0,6	↑
ACS (LE)		2	F	34	4,0	1,9	2,1	↑
JRM (RE)	23				5,0	3,9	1,1	↑
JRM (LE)		2	F	34	3,9	3,9	0	↓
AEN (RE)	24				5,3	3,1	2,2	↑
AEN (LE)		3	F	40	3,6	1,4	2,2	↑
TFZ (RE)	25				7,4	6,5	0,9	↑
TFZ (LE)		2	F	32	8,3	6,0	2,3	↑
EMP (RE)	26				12,2	11,6	0,6	↑
EMP (LE)		2	F	34	7,1	6,1	1,0	↑
ARBA (RE)	27				5,6	4,5	1,1	↑
ARBA (LE)		2	F	37	5,1	5,1	0	↓
VVO (RE)	28				9,4	9,2	0,2	↑
VVO (LE)		2	F	36	8,2	5,5	2,7	↑
RMMG (RE)	29				2,8	2,3	0,5	↑
RMMG (LE)		3	F	40	4,4	4,1	0,3	↑
MFCM (RE)	30				1,2	10,8	1,2	↑
MFCM (LE)		2	F	34	10,7	9,9	0,8	↑
ERA (RE)	31				3,2	2,6	0,6	↑
ERA (LE)		4	M	50	3,1	2,8	0,3	↑
JTA (RE)	32				*	*	*	
JTA (LE)		3	M	46	*	*	*	
ABA (RE)	33				2,4	2,8	-0,4	↓
ABA (LE)		3	F	46	1,4	1,2	0,2	↑
ZB (RE)	34				5,8	///	5,8	↑
ZB (LE)		3	F	41	1,9	2,4	-0,5	↓
MB (RE)	35				3,9	3,4	0,5	↑
MB (LE)		3	F	42	3,7	3,9	-0,2	↓
PM (RE)	36				8,6	8,6	0	↓
PM (LE)		3	F	42	4,8	3,6	1,2	↑
EN (RE)	37				*	*	*	
EN (LE)		3	F	49	*	*	*	
EAS (RE)	38				1,8	1,8	0	↓
EAS (LE)		3	F	41	3,1	3,6	-0,5	↓
ASM (RE)	39				*	*	*	
ASM (LE)		3	F	42	*	*	*	
MCD (RE)	40				*	*	*	
MCD (LE)		3	F	41	0,6	-1,1	1,7	↑
NBS (RE)	41				*	*	*	
NBS (LE)		4	F	50	5,5	4,3	1,2	↑
JAS (RE)	42				8,2	7,6	0,6	↑
JAS (LE)		3	F	42	6,0	5,7	0,3	↑
MF (RE)	43				*	*	*	
MF (LE)		3	F	49	4,9	4,8	0,1	↑
RR (RE)	44				8,3	7,6	0,7	↑
RR (LE)		3	F	44	4,3	4,1	0,2	↑
SI (RE)	45				1,2	0	1,2	↑
SI (LE)		3	F	43	*	*	*	
LTZS (RE)	46				8,5	7,7	0,8	↑
LTZS (LE)		4	F	53	4,0	3,2	0,8	↑
ECA (RE)	47				*	*	*	
ECA (LE)		4	F	59	*	*	*	
MAS (RE)	48				2,8	0,1	2,7	↑
MAS (LE)		4	F	53	*	*	*	
EML (RE)	49				6,5	6,5	0	↓
EML (RE)		4	F	53	5,4	5,2	0,2	↑
CAS (RE)	50				6,3	5,9	0,4	↑
CAS (LE)		4	M	59	9,8	9,6	0,2	↑
LF (RE)	51				1,2	1,4	-0,2	↓
LF (LE)		4	F	58	2,0	2,5	-0,5	↓
MTB (RE)	52				1,6	1,7	-0,1	↓
MTB (LE)		4	F	52	1	0,1	0,9	↑
JCO (RE)	53				1,7	2,3	-0,6	↓
JCO (LE)		4	M	58	1,6	///	1,6	↑
TJO (RE)	54				*	*	*	
TJO (LE)		4	F	56	*	*	*	
RAC (RE)	55				11,8	11,6	0,2	↑
RAC (LE)		4	F	53	*	*	*	
OAS (RE)	56				1,6	1,4	0,2	↑
OAS (LE)		4	M	59	0,7	2	-1,3	↓
ZCL (RE)	57				*	*	*	
ZCL (LE)		4	F	59	0,8	0,9	-0,1	↓
MAP (RE)	58				*	*	*	
MAP (LE)		4	F	52	*	*	*	
IV (RE)	59				*	*	*	
IV (LE)		4	M	53	*	*	*	
MMG (RE)	60				10,2	9,3	0,9	↑
MMG (LE)		4	F	59	5,8	6,3	-0,5	↓
IM (RE)	61				4,9	3,6	1,3	↑
IM (LE)		5	F	64	3,9	3,0	0,9	↑
RMM (RE)	62				3,0	1,3	1,7	↑
RMM (LE)		5	F	72	0,6	0,7	-0,1	↓
VRF (RE)	63				0,8	0,4	0,4	↑
VRF (LE)		5	F	64	4,3	2,8	1,5	↑
VR (RE)	64				*	*	*	
VR (LE)		5	F	73	*	*	*	
ZDG (RE)	65				4,4	3,9	0,5	↑
ZDG (LE)		5	F	64	3,6	4,9	-1,3	↓
SK (RE)	66				3,1	3,5	-0,4	↓
SK (LE)		5	F	60	2,1	1,6	0,5	↑
RY (RE)	67				0,2	///	0,2	↑
RY (LE)		5	F	60	2,8	1,1	1,7	↑
LNS (RE)	68				8,0	8,4	-0,4	↓
LNS (LE)		5	F	67	11,8	11,5	0,3	↑
CCP (RE)	39				0,7	2,7	-2,0	↓
CCP (LE)		5	F	68	*	*	*	
AM (RE)	70				1,0	0,8	0,2	↑
AM (LE)		5	F	63	1,4	2,1	-0,7	↓
ACL (RE)	71				2,8	2,6	0,2	↑
ACL (LE)		5	M	62	3,8	3,8	0	↓
EG (RE)	72				*	*	*	
EG (LE)		5	F	62	*	*	*	
SM (RE)	73				2,3	2,8	-0,5	↓
SM (LE)		5	M	69	2,2	1,7	0,5	↑
CA (RE)	74				*	*	*	
CA (LE)		5	F	72	2,6	2,9	-0,3	↓
DO (RE)	75				2,4	2,8	-0,4	↓
DO (LE)		5	M	65	1,7	2,3	-0,6	↓

* Absent TEOAE responses; ↑ Presence of the TEOAE reduction/suppression effect; ↓ Absence of the TEOAE reduction/suppression effect; NN: no noise. WN: with noise.

Double factor analysis of variance without repetitions- alpha (significance level) equaling 0.05 (5%) - for a condition of absent noise, comparing the right and left ears, revealed that there was no statistically significant difference, as shown in Table 3.

**Table 3:** Analysis of variance for the condition of absent noise, comparing the right and left ears

ANOVA						
Source of variation	SQ	gl	MQ	F	*p*-value	critical F
Lines	907,3449	54	16,80268	3,322176	9,8E-06	1,570884
Columns	8,127364	1	8,127364	1,606918	0,210365	4,019541
Error	273,1176	54	5,057734			
Total	1188,59	109				

Key: SQ: sum of squares of deviations; gl: degrees of freedom for the distribution SQ = sum of squares; MQ = Squares mean, F = variance among groups, *p* = probability and critical F = critical factor

Again, double factor analysis of variance without repetitions- alpha (significance level) equaling 0.05 (5%) - for a condition of noise, comparing the right and left ears, revealed that there was no statistically significant difference, as shown in Table 4.

**Table 4:** Analysis of variance for the condition of noise, comparing the right and left ears

ANOVA						
Source of variation	SQ	Gl	MQ	F	*p*-value	critical F
Lines	360,9992	24	15,04163	6,177178	1,6E-05	1,98376
Columns	2,4642	1	2,4642	1,011978	0,324465	4,259677
Error	58,4408	24	2,435033			
Total	421,9042	49				

Table 5 presents the double factor analysis of variance without repetitions- alpha (significance level) equaling 0.05 (5%) - for the degree of reduction, comparing the right and left ears, revealed that there was no statistically significant difference between both ears.

**Table 5:** Analysis of variance for the degree of reduction, comparing right and left ears

ANOVA						
Source of variation	SQ	gl	MQ	F	*p*-value	critical F
Lines	66,83119	58	1,152262	1,518049	0,057397	1,545768
Columns	0,030593	1	0,030593	0,040305	0,841588	4,006873
Error	44,02441	58	0,759041			
Total	110,8862	117				

## DISCUSSION

A comparison of our findings (presented in Annex 1 and treated statistically - Tables 3, 4 and 5) with studies by Khalfa & Collet^7^, and Khalfa et al.^8^, who applied similar methods, shows that the findings did not correlate. In our study, there was no statistically significant difference for the presence of the suppression/reduction effect of evoked otoacoustic emissions and its amplitude relative to right/left dominance in normal hearing adult subjects. Those authors documented differences in patterns of suppression/reduction of evoked otoacoustic emissions in the right and left ears.

The results of those authors indicated interaural asymmetry, not only relative to the amplitude of evoked otoacoustic emissions, which was higher on the right, but also the action of the efferent auditory system, which was also more effective on the right^7^,^8^. According to those authors, such difference could also explain TEOAE asymmetry - lateralization of auditory sensitivity and temporary changes in thresholds and tinnitus. Those authors, however, did not explain the reasons for their findings, but highlighted the need for further studies to investigate the asymmetry^7^.

Studies on suppression of otoacoustic emissions that aim to investigate differences in laterality are sparse; existing studies use a wide range of methods, which complicates comparisons. Thus, additional studies are needed for new comparisons and discoveries.

Several studies have demonstrated a range of parameters for recording otoacoustic emissions and the suppression effect of otoacoustic emissions adequately. These findings help organize methods and have shown several features such as: the response amplitude analysis interval; the intensity of contralateral noise versus suppression amplitude; and the bilateral mode for recording compared with ipsilateral or contralateral modes and time intervals^1^. However, there is a gap in the factor sex and right/left ear lateralization.

The physiology and anatomy specifically of the medial efferent olivocochlear tract is poorly known, which underlines the need for more detailed knowledge about the implications of a possible lateral dominance of reduction/suppression of otoacoustic emissions.

## CONCLUSION

This study found no differences in patterns between right and left ears relative to amplitude asymmetry, and the presence of the TEOAE suppression/reduction effect in the study sample; these results differ from other papers that were reviewed.

## References

[1] Hood LJ, Berlin CI, Goforth-Barter L, Wen H., Berlin CI. (1999). The efferent auditory system. Basic science and clinical applications..

[2] Parthasarathy TK. (2001). Aging and contralateral suppression effects on transient evoked otoacoustic emissions.. J Am Acad Audiol..

[3] Hood LJ, Berlin CI., Sininger Y, Starr A. (2001). Auditory neuropathy: a new perspective on hearing disorders..

[4] Breuel MLF, Sanchez TG, Bento RF. (2001). Vias auditivas eferentes e seu papel no sistema auditivo.. Arq Int Otorrinolaringol..

[5] Azevedo MF., Figueiredo MS. (2003). Conhecimentos para entender bem as emissões otoacústicas e BERA..

[6] Sahley TL, Nodar RH, Musiek FE. (1997). Efferent auditory system- Structure and function..

[7] Khalfa S, Collet L. (1996). Functional asymmetry of medial olivocochlear system in humans. Towards a peripheral auditory lateralization.. Neuroreport..

[8] Khalfa S, Micheyl C, Veuillet E, Collet L. (1998). Peripheral auditory lateralization assessment using TEOAEs.. Hear Res..

[9] Glattke TJ, Robinette MS., Glattke TJ, Robinette MS. (2002). Otoacoustic emissions: clinical applications..

[10] Verbeke G, Molenberghs G. (1997). Linear mixed models in practice..

